# Long Term Streptomycin Toxicity in the Treatment of Buruli Ulcer: Follow-up of Participants in the BURULICO Drug Trial

**DOI:** 10.1371/journal.pntd.0002739

**Published:** 2014-03-13

**Authors:** Sandor Klis, Ymkje Stienstra, Richard O. Phillips, Kabiru Mohammed Abass, Wilson Tuah, Tjip S. van der Werf

**Affiliations:** 1 Department of Internal Medicine, Infectious Disease Service, University Medical Center Groningen, Groningen, the Netherlands; 2 Komfo Anokye Teaching Hospital, Kumasi, Ghana; 3 Agogo Presbyterian Hospital, Agogo, Ghana; 4 Nkawie-Toase Government Hospital, Nkawie, Ghana; 5 Department of Pulmonary Medicine and Tuberculosis, University Medical Center Groningen, Groningen, the Netherlands; Fondation Raoul Follereau, France

## Abstract

**Background:**

Buruli Ulcer (BU) is a tropical infectious skin disease that is currently treated with 8 weeks of intramuscular streptomycin and oral rifampicin. As prolonged streptomycin administration can cause both oto- and nephrotoxicity, we evaluated its long term toxicity by following-up former BU patients that had received either 4 or 8 weeks of streptomycin in addition to other drugs between 2006 and 2008, in the context of a randomized controlled trial.

**Methods:**

Former patients were retrieved in 2012, and oto- and nephrotoxicity were determined by audiometry and serum creatinine levels. Data were compared with baseline and week 8 measurements during the drug trial.

**Results:**

Of the total of 151 former patients, 127 (84%) were retrieved. Ototoxicity was present in 29% of adults and 25% of children. Adults in the 8 week streptomycin group had significantly higher hearing thresholds in all frequencies at long term follow-up, and these differences were most prominent in the high frequencies. In children, no differences between the two treatment arms were found. Nephrotoxicity that had been detected in 14% of adults and in 13% of children during treatment, was present in only 2.4% of patients at long term follow-up.

**Conclusions:**

Prolonged streptomycin administration in the adult study subjects caused significant persistent hearing loss, especially in the high frequency range. Nephrotoxicity was also present in both adults and children but appeared to be transient. Streptomycin should be given with caution especially in patients aged 16 or older, and in individuals with concurrent risks for renal dysfunction or hearing loss.

## Author Summary

Buruli Ulcer is an infectious skin disease, mainly occurring in West Africa. Previously, the disease was treated exclusively by surgery, but in the last decade, effective treatment with antibiotics has been established. The WHO recommended regimen consists of 8 weeks of oral rifampicin combined with intramuscular streptomycin. However, prolonged use of streptomycin is known to cause permanent ototoxicity and transient nephrotoxicity. To study this, we performed audiometry and measured the serum creatinine in 127 former Buruli ulcer patients who received either 4 or 8 weeks of streptomycin 4 to 6 years ago. Ototoxicity was present in 29% of adults and 25% of children. Adults who received 8 weeks of streptomycin had significantly worse hearing at long term follow-up, and this was most prominent in the high frequencies. In children, no differences between the two groups were found. Nephrotoxicity that had been detected in 14% of adults and in 13% of children during treatment, was present in only 2.4% of patients at long term follow-up. The findings indicate that caution should be exercised when prescribing streptomycin to adults for prolonged periods of time. Treatment regimens for Buruli ulcer that do not contain streptomycin are desirable and should be investigated.

## Introduction

Buruli ulcer (BU) is a Neglected Tropical Disease caused by infection with *Mycobacterium ulcerans*. Necrotic skin lesions characterize the disease, often with typical undermined edges. Treatment delay may result in disfigurement and functional limitations [1]. Though isolated cases or small outbreaks have been reported in over 30 countries, the disease is mainly found in endemic areas in rural West- and Central-Africa, with around 6.000 cases occurring annually [2]–[4].

Over the past decade the main mode of treatment has shifted from surgery to antimicrobial therapy. Several antibiotic regimens have been proposed and studied, and the current regimen advised by the World Health Organization consists of 8 weeks of oral rifampicin and intramuscular streptomycin [5]–[8]. Although surgery can be problematic in under-resourced developing countries due to high costs and low availability, prolonged streptomycin administration has complications of its own. These complications are partly related to the parenteral mode of administration of streptomycin, but also to its intrinsic toxicity, notably, ototoxicity and nephrotoxicity [9].

Streptomycin induced ototoxicity encompasses damage to both the cochlea and vestibulum [10], although the effects of vestibular damage can to some extent be compensated for by the brain, and so are clinically less prominent [11]. The damage is caused by reactive oxygen species that are formed by a redox-active complex of parts of the aminoglycoside and biologically available iron ions [12]. The most frequently reported manifestations of ototoxicity are high-frequency sensorineural hearing loss, and vestibular dysfunction resulting in disequilibrium and loss of the vestibular ocular-reflex [12]. These side-effects are often transient, but chronic effects, especially hearing loss, have also been reported. The wide range in incidence varies between 5% and 25%, depending on the specific aminoglycoside drug, patient group (older age groups are probably more vulnerable), and definition of ototoxicity including the method of detection [13], [14]. The occurrence of ototoxicity does not appear to be related to the individual dosages or the frequency of administration per se, but rather to the cumulative dose, and the age of the patient [15]. Also, there appears to be a modest genetic susceptibility, caused by a point mutation in mitochondrial DNA [16].

The nephrotoxic effects of streptomycin are most prominent in the proximal tubule, and transient nephropathy, manifested by a slow rise in serum creatinine and a decreased glomerular filtration rate, is observed in 10–20% of therapeutic courses [17]. Unlike aminoglycoside-induced ototoxicity, long-term kidney damage is uncommon. It has long been hypothesized that aminoglycoside-induced nephrotoxicity would lead to increased serum concentrations of the drug, resulting in ototoxicity, or that some other link between aminoglycoside induced oto- and nephrotoxicity existed. Several studies have however been unable to demonstrate such a relationship, and it is believed that the two are largely independent [15], [18], [19].

With a total cumulative dose of up to 56 g (15 mg/kg daily with a 1 g daily maximum, 56 doses), the amount of streptomycin that is administered to treat BU is higher than in the treatment of brucellosis, endocarditis, and urinary tract infections, but similar to the treatment of *Mycobacterium avium* complex and second line treatment for tuberculosis. However, no data exist on the long-term toxicity of the currently recommended regimen of antimicrobial therapy in BU. Therefore, we chose to study former BU patients that participated earlier in a randomized controlled trial conducted between 2006 and 2008 [20]. In that trial patients were randomized to receive either 8 or 4 weeks of streptomycin, allowing a direct comparison between the two groups in terms of long-term streptomycin toxicity.

## Materials and Methods

### Ethics

The study protocol was approved by the Committee on Human Research, Publication, and Ethics of the Kwame Nkrumah University of Science and Technology and the Komfo Anokye Teaching Hospital, Kumasi (reference number CHRPE/AP/133/12). Written informed consent was obtained from all participants aged ≥12 years, and consent from parents, or legal representatives of participants aged ≤18 years.

### Sample size and patient recruitment

We retrieved our study subjects among individuals that had earlier participated in the Burulico trial, conducted between 2006 and 2009 in Ghana, registered with number NCT00321178 at clinicaltrials.gov. For that trial, patients aged 5 years or older, clinically diagnosed with early (duration <6 months), limited (cross-sectional diameter <10 cm) *M ulcerans* infection were included, and randomized to receive either 8 weeks of streptomycin at 15 mg/kg daily (max 1000 mg daily) and 8 weeks of rifampicin at 10 mg/kg daily (max 600 mg daily), or 4 weeks of streptomycin and rifampicin, followed by 4 weeks of rifampicin and clarithromycin at 7.5 mg/kg daily. Pregnancy, drug intolerance, and renal, hepatic, and acoustic impairment were among the exclusion criteria, and co-medication with drugs other than the study drugs occurred in less than 5% of patients, and mainly consisted of NSAIDs. Patients had a median age of 12 and 30% were male. Patient characteristics are described in more detail in Nienhuis et al. [20].

For the present follow-up study, participants were retrieved between June and November 2012 by visiting their last known village or through telephone contact if available. If the former patient was no longer living at the last known village, neighbors, relatives, and community leaders were asked for additional information. When a former patient was located, he or she was informed about the study, given time to consider participation, and was asked for consent.

### Procedures

The equipment and procedure followed for audiometry at follow-up were identical to those during the drug trial. Audiometry was performed in a quiet room in one of the two study hospitals of the BURULICO drug trial with portable Interacoustics AS208 audiometers, with circumaural earphones with noise reducing Peltor mute cushions. The audiometers were calibrated to ISO64, both before the drug trial and again before the follow-up. Biological calibration performed weekly. During a biological calibration session, a person with known, stable hearing thresholds was tested. A persistent deviation of 10 dB or more would prompt acoustic calibration of the audiometer. However, both during the drug trial and the follow-up study, no acoustic calibration was necessary. As soundproof rooms were not available at the study sites, ambient noise (AN) levels were measured with a voltcraft SL-100 ambient noise meter in dBA filter weighting during each audiometry session.

The person performing the follow-up audiometry was blinded to the treatment allocation during the Burulico study, and was therefore unaware of earlier streptomycin treatment duration. Both ears were tested, the right ear first. Frequencies were tested in the following order: 1, 2, 4, 6, 8, 0.5 and 0.25 kHz. Testing started with presenting a loud tone. Then the intensity was decreased in steps of 5 dB until the tone was not heard anymore. The last tone still heard was the level of audibility of the first test run. Then the intensity was increased with 10 dB before being decreased again in steps of 5 dB, until the tone was no longer heard (the second test run). If the results of the two test runs were not identical, the process was repeated until two identical levels of audibility were found. This level was considered the hearing threshold. The same procedure was followed during the drug trial, and so, audiograms were available for baseline, after 8 weeks of treatment, and at long-term follow-up. In addition to audiometry, each subject was asked at follow-up about complaints of hearing loss and dizziness after completion of treatment.

To detect late nephrotoxic effects of the streptomycin administration 4–6 years earlier, serum creatinine was measured in a sample of venous blood (approximately 2 ml) at the same laboratory, where serum creatinine was measured during the drug trial, using the same analytic technique. The results were compared with the measurements at baseline, after 2, 4, 6, and 8 weeks of treatment during the BURULICO study. Patients with suspected long-term nephrotoxicity were referred to the hospital for further management.

The total cumulative dose of streptomycin administered was estimated to be 56 times the daily dose. Individual records of compliance were not available, but during the drug trial, streptomycin administration was only performed in participating hospitals and health centers and was recorded on tally sheets with compliance approaching 100%.

### Data analysis

As aminoglycoside toxicity is reported to occur less frequently in children [21], [22], we analyzed the results separately for those ≤16 and >16 years of age at the time of receiving streptomycin treatment during the Burulico trial. For each frequency, the mean of measurement results of audiometry of both ears was recorded. As the distribution of hearing thresholds is usually skewed to the left, differences between the two treatment arms were assessed with nonparametric Mann-Whitney-U tests. According to the criteria of the American Speech-Language-Hearing Association [23], an increased threshold of 20 dB at any one frequency or an increase of 15 dB at two consecutive frequencies was classified as hearing loss. Nephrotoxicity was defined as a rise in serum creatinine at two consecutive assessments during treatment compared to baseline of ≥44 µmol/L (0.5 mg/dL) or 50% [17], [24]. As serum creatinine levels increase with age and body weight, especially muscle mass, it was not possible to make a meaningful direct comparison between the serum creatinine concentrations in the present study with those obtained 4–6 years earlier during the drug trial. Instead, a decrease of the estimated Glomerular Filtration Rate (eGFR) of >25% in this follow-up study compared to baseline was defined as long term nephrotoxicity [25]. In adults, the eGFR was calculated using the Chronic Kidney Disease Epidemiology Collaboration (CKD-EPI) equation [26], as this was earlier shown to be superior to other equations in a Ghanaian population [27]. In children the eGFR was computed using the revised Schwartz formula [28].

## Results

### Patient population and retrieval

Of the 151 former participants of the Burulico trial 127 individuals (84%) were retrieved for follow up. Although the trial had taken place in the Ashanti region of Ghana, many former patients were not ethnically Ashanti, and had moved away from the study site, and patients were retrieved in 9 of Ghanas 11 regions, including the three Northern regions more than 700 kilometers and approximately 10 hours by road from the original study site. 68% of the retrieved former patients were female, and the median age at follow-up was 18 years. Sixty-eight percent of our study participants had been 16 or younger at the time they received treatment during the Burulico trial. Of the 24 former patients not retrieved, 4 were already lost to follow-up during the Burulico trial, 3 had moved abroad, 2 had deceased, and the fate of the remaining 15 was unknown. The patients that were lost to follow-up did not differ significantly from the patients that were retrieved in terms of baseline and 8 week audiometry and serum creatinine, age, sex or treatment arm. The median duration between drug administration and follow-up was 5 years. Overall, the patients reported to be doing well. 28% reported to have experienced another disease since their BU was cured, with most patients reporting malaria, headache, and stomach ache. One retrieved patient was HIV-positive. Three patients reported currently taking analgesics, and 2 patients reported currently taking anti-hypertensives.

### Ototoxicity

**Figure 1:**
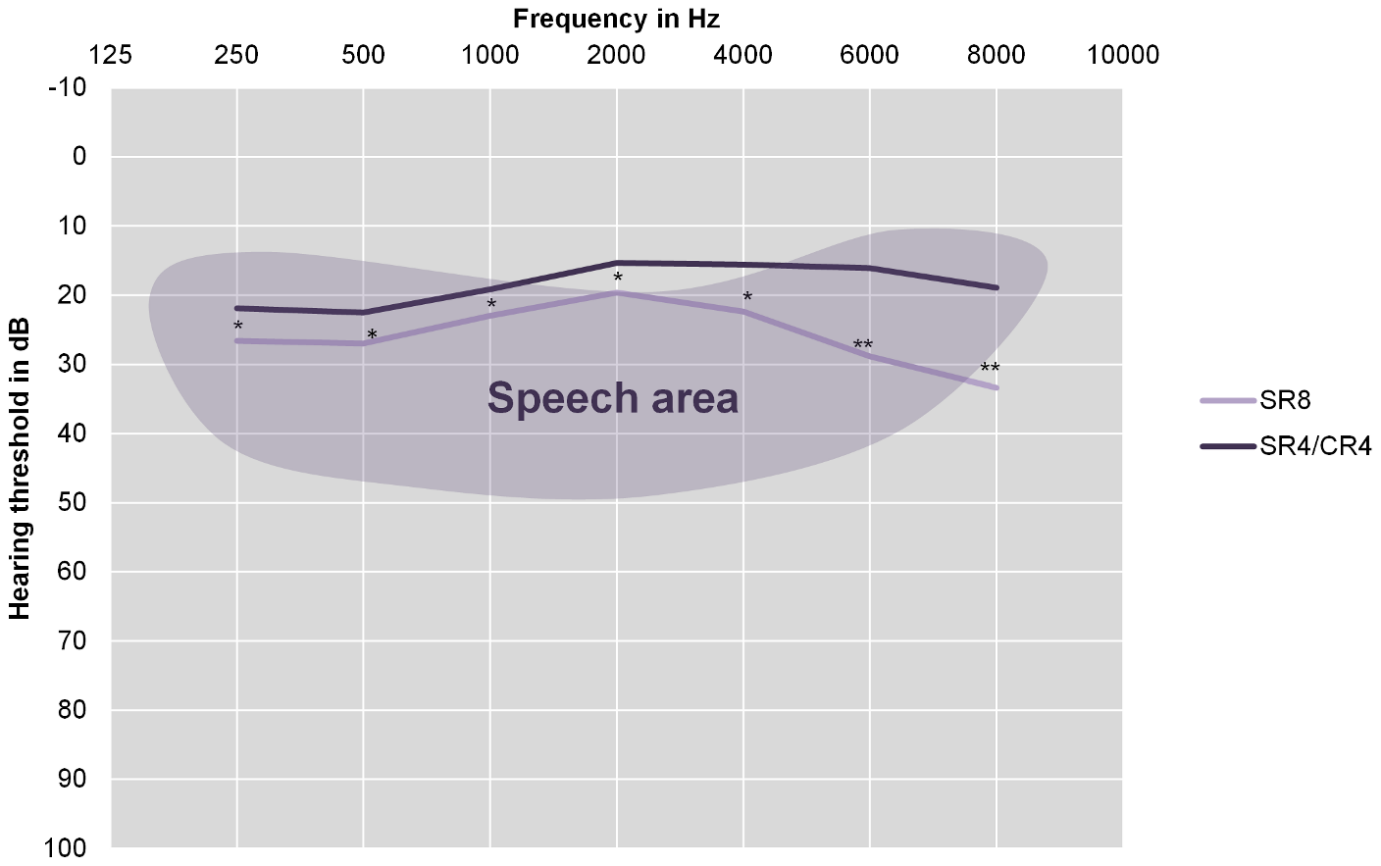
Hearing thresholds of adults at long term follow-up. Mean hearing thresholds of adults (16 or older at the time of treatment) in dB averaged over both ears. The shaded area represents the area used in understanding human speech (Pascoe, 1980) [37]. SR8 = participants in the 8 week Streptomycin/Rifampicin group. SR4/CR4 = participants in the 4 week Streptomycin/Rifampicin plus 4 week Clarithromycin/Rifampicin group. The Differences between the two groups were tested with the Mann-Whitney U test, 1-tailed. * = p<0.05, ** = p<0.001.

**Figure 2:**
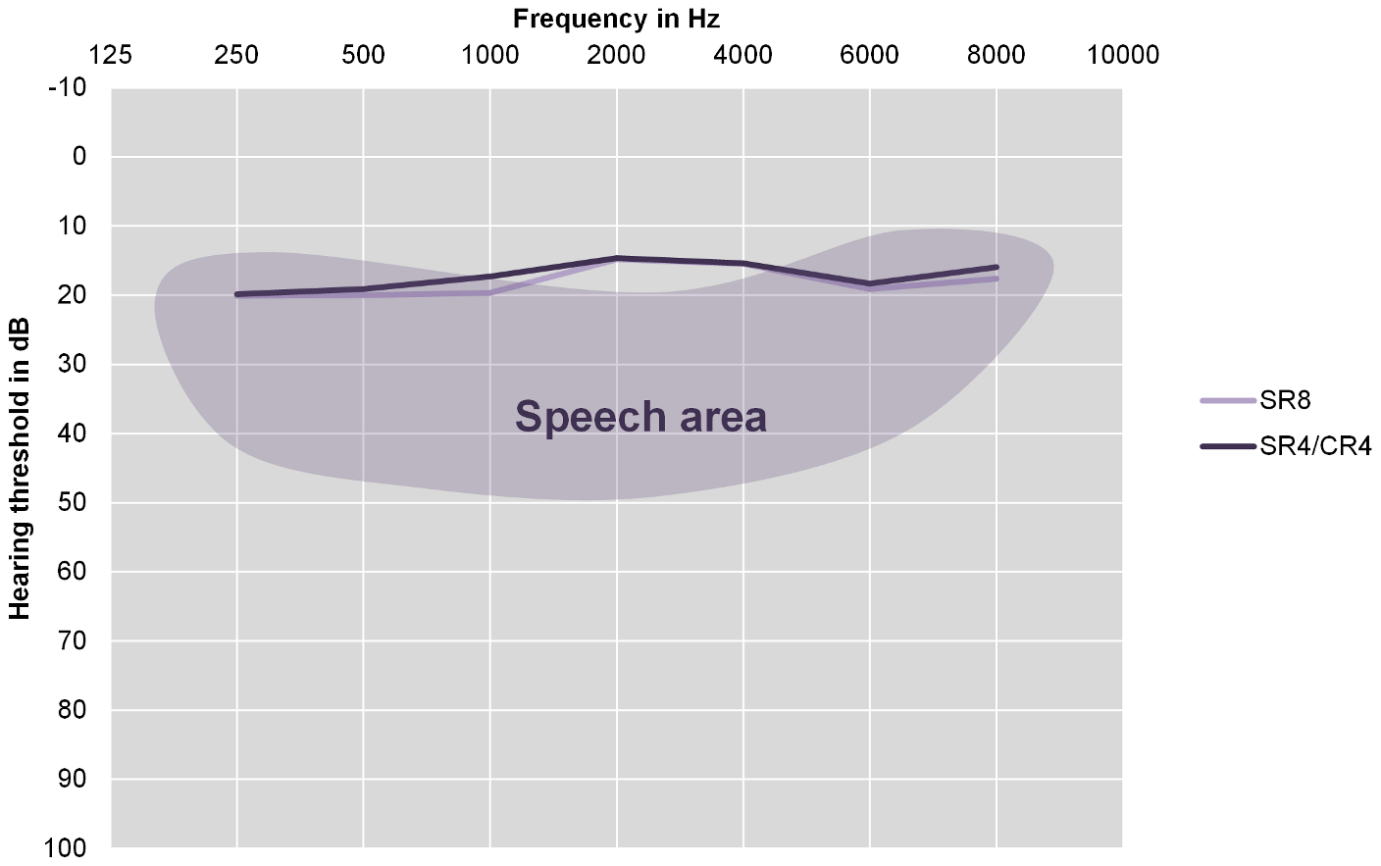
Hearing thresholds of children at long term follow-up. Mean hearing thresholds of children (under 16 at the time of treatment) in dB averaged over both ears. The shaded area represents the area used in understanding human speech (Pascoe, 1980) [37]. SR8 = participants in the 8 week Streptomycin/Rifampicin group. SR4/CR4 = participants in the 4 week Streptomycin/Rifampicin plus 4 week Clarithromycin/Rifampicin group. The Differences between the two groups were tested with the Mann-Whitney U test, all differences were ns.

Twenty-nine percent of adults, and 25% of children were classified as having hearing loss compared to baseline audiometry after 8 weeks of treatment, and 31% of adults, and 27% of children were classified as having hearing loss at long term follow-up compared to baseline audiometry. Hearing loss after 8 weeks was significantly associated with hearing loss at long term follow-up in (*p* = 0.017 by *Χ^2^*), but not in children (*p* = 0.102 by *Χ^2^*). The average hearing thresholds at long-term follow up for both treatment arms for adults are shown in Figure 1, and for children in Figure 2. For adults, no differences between the two treatment arms existed in any of the frequencies at baseline, and after 8 weeks of treatment, there was a significant difference in hearing threshold between treatment arms at 6000 Hz (*p* = 0.03) in adults, while those at 500 (*p* = 0.07), 1000 (*p* = 0.09), and 8000 Hz (*p* = 0.09) approached significance by Mann-Whitney U test. For children, there were no significant differences between the treatment arms at any frequency at baseline or after 8 weeks of treatment. For both adults and children, univariate associations between long term ototoxicity and treatment arm, age, sex, total cumulative dose of streptomycin and complaints of hearing loss were determined. The results are shown in table 1. The average (SD) level of ambient noise (AN) was 45.6 (6.5) dB.

**Table 1:** Univariate associations with long term ototoxicity.

*Adults* (*N* = 41)	Ototoxicity		
	Yes	No	*P* value
Treatment arm			
8 weeks streptomycin	40%	60%	
4 weeks streptomycin	23%	77%	.114
Age during treatment (SD)	32(17)	27(11)	.150
Sex			
Male	57%	43%	
Female	26%	74%	.101
Total grams of streptomycin (SD)	40(15)	34(13)	.090
Complaints of hearing loss			
Yes	25%	75%	
No	32%	68%	.393

*Children*(*N* = 86)	Ototoxicity		
	Yes	No	*P* value
Treatment arm			
8 weeks streptomycin	26%	74%	
4 weeks streptomycin	28%	72%	.414
Age during treatment (SD)	10(3)	9(3)	.274
Sex			
Male	24%	76%	
Female	29%	71%	.505
Total grams of streptomycin (SD)	23(12)	21(9)	.220
Complaints of hearing loss			
Yes	40%	60%	
No	25%	75%	.164

All *P* values are 1-tailed except for sex.

From self-report, 10% of adults reported experiencing hearing loss (5% in the 8 week streptomycin group vs 16% in the 4 week streptomycin group; *p*>0.3 by *Χ^2^*) and 12% of children reported experiencing hearing loss (9% in the 8 week streptomycin group vs 15% in the 4 week streptomycin group; *p*>0.3 by *Χ^2^*) after treatment was completed. Ten percent of adults reported experiencing dizzyness (5% in the 8 week streptomycin group vs 16% in the 4 week streptomycin group; *p*>0.3 by *Χ^2^*) and 12% of children reported experiencing dizzyness (9% in the 8 week streptomycin group vs 15% in the 4 week streptomycin group; *p*>0.3 by *Χ^2^*) after treatment was completed. There appeared to be no association between audiometrically classified and self-reported hearing loss, as 14% of the patients that were audiometrically classified as having hearing loss at long term follow-up also complained of hearing loss vs 9% of patients that were not audiometrically classified as having hearing loss (*p*>0.3 by *Χ^2^*).

### Nephrotoxicity

**Figure 3:**
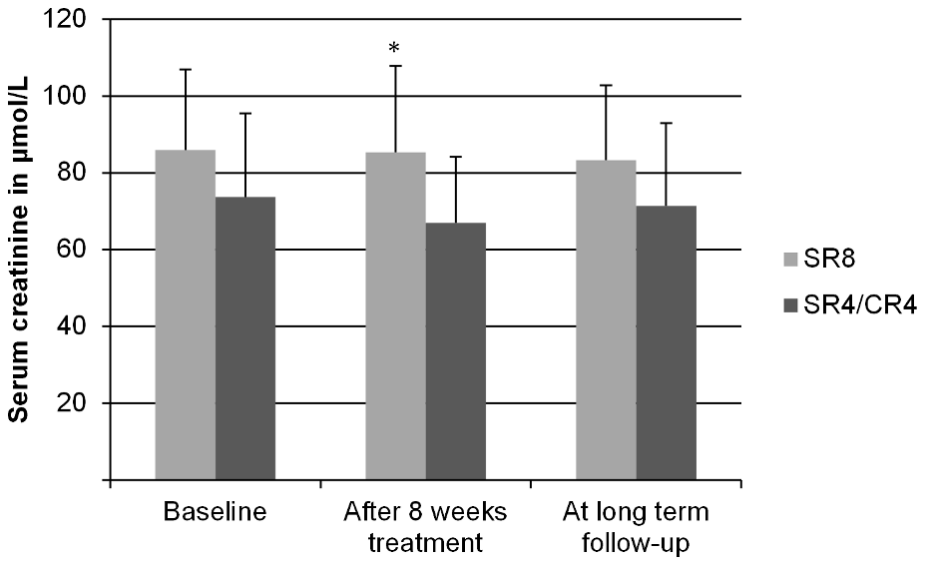
Serum creatinine levels in adults at long term follow-up. Mean (SD) serum creatinine concentrations of adults (16 or older at the time of treatment), measured at baseline, after 8 weeks of treatment and at long term follow-up. SR8 = participants in the 8 week Streptomycin/Rifampicin group. SR4/CR4 = participants in the 4 week Streptomycin/Rifampicin plus 4 week Clarithromycin/Rifampicin group. The differences between the two groups at baseline were tested with the students t-test. The differences between the two groups after 8 weeks of treatment and at long-term follow up were tested with an ANCOVA, controlling for serum creatinine concentration at baseline and age. * = *p*<0.05, others ns (*N* = 41).

**Figure 4:**
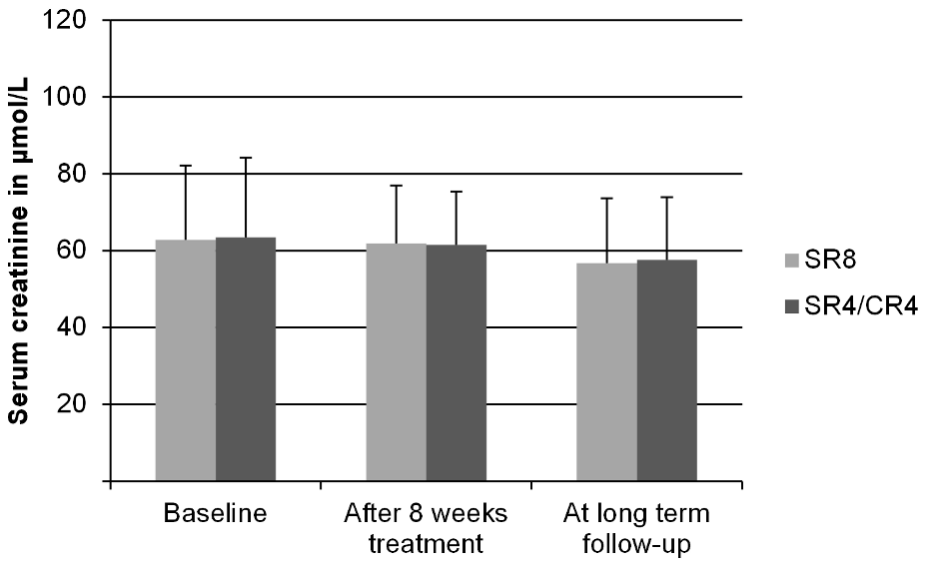
Serum creatinine levels in children at long term follow-up. Mean (SD) serum creatinine concentrations of children (under 16 at the time of treatment), measured at baseline, after 8 weeks of treatment and at long term follow-up. SR8 = participants in the 8 week Streptomycin/Rifampicin group. SR4/CR4 = participants in the 4 week Streptomycin/Rifampicin plus 4 week Clarithromycin/Rifampicin group. The differences between the two groups were tested with the students t-test. All differences ns (*N* = 84).

During treatment, 14% of adults, and 13% of children were classified as having nephrotoxicity. At long term follow-up 1 adult (2.4%) and 2 children (2.4%) were classified as having long-term nephrotoxicity. All 3 of these patients had received streptomycin for 8 weeks (*p*<0.1 by *Χ^2^*), but only 1 was also classified as having nephrotoxicity during treatment. The average serum creatinine concentrations for both treatment arms for adults are shown in Figure 3, and for children in Figure 4. For both adults and children, univariate associations between nephrotoxicity and treatment arm, age, weight, sex, and total cumulative dose of streptomycin were determined. The results are shown in table 2.

**Table 2:** Univariate associations with nephrotoxicity during treatment.

*Adults* (*N* = 41)	Nephrotoxicity		
	Yes	No	*P* value
Treatment arm			
8 weeks streptomycin	20%	80%	
4 weeks streptomycin	9%	91%	.157
Age during treatment (SD)	29(10)	29(13)	.470
Sex			
Male	29%	71%	
Female	11%	89%	.237
Total grams of streptomycin (SD)	40(11)	35(15)	.178

Children (*N* = 86)	Nephrotoxicity		
	Yes	No	*P* value
Treatment arm			
8 weeks streptomycin	20%	80%	
4 weeks streptomycin	5%	95%	.024
Age during treatment (SD)	10(3)	9(3)	.254
Sex			
Male	9%	91%	
Female	16%	84%	.356
Total grams of streptomycin (SD)	26(7)	21(10)	.041

All *P* values are 1-tailed except for sex.

## Discussion

In this study we followed-up individuals that were randomly assigned to either 4 or 8 weeks of streptomycin injections 4 to 6 years earlier, and that had similar results in audiometry at baseline. This provided a unique model to compare long-term toxicity incurred by doubling the cumulative dosage of streptomycin. In adults, we found an important and clinically relevant increase in hearing loss in the high frequencies in individuals that had received 8 weeks of streptomycin, in comparison to those that had received only 4 weeks of this aminoglycoside. In children, no differences between the two treatment arms were detectable.

Although macrolides, especially, azithromycin have also been connected to hearing loss [29], the ototoxicity associated with prolonged streptomycin use was significantly more pronounced than in individuals that had received clarithromycin instead.

The ototoxic effects of prolonged duration of streptomycin therapy appeared to differ between adults and children. To our knowledge, no studies have evaluated the ototoxicity of an aminoglycoside in a study population consisting of both children and adults suffering from the same illness, exposed to the same treatment schedule. Low levels of ototoxicity in children have been reported before [21], [22], but the exact mechanisms for this interaction with age are not well understood [21].

The incidence of hearing loss in our study was slightly higher than in earlier reports, which may be due to the fact that we used the formal ASHA criteria that are designed to detect early changes, so as to avoid more severe damage with ongoing aminoglycoside administration.

In children, there was only a weak relationship between hearing loss detected by audiometry and complaints of hearing loss by the patient, and in adults there was no relationship at all. Audiometric and subjective ototoxicity often correlate poorly [15], [30], [31], because audiometric toxicity is usually – as in our study – present in the high frequencies. High frequency hearing loss does not affect primary speech hearing, but rather affects the ability to follow group conversations, and causes people to hear the wrong words without noticing it [32]. This may explain why many clinicians are not very concerned about ototoxicity in the treatment of BU, as their patients usually do not report hearing loss spontaneously.

In our study, nephrotoxicity was present in both children and adults, occurred more often in the 8 week streptomycin group and appeared to be related to the total cumulative dose of streptomycin. However, long term nephrotoxicity was only detected in 3 study subjects. The occurrence of both transient and long-term streptomycin nephrotoxicity in our study might be a low estimate for the population at risk in general, as BU is a local infection, usually without systemic involvement, and most patients were young and did not have any significant comorbidities, medical history or co-medication, contrary to other patient categories that are administered aminoglycosides, e.g. ICU patients or those with cystic fibrosis. In addition, streptomycin was administered once daily, which is known to reduce the incidence of nephrotoxicity [33].

Our study had several limitations. First, after an intensive search, we only retrieved 127 (84%) of the population that we intended to study. Of the 24 subjects that were lost to follow-up, we could not detect features that might have introduced bias, but we cannot completely rule out that some bias was introduced. Next, although the group that was earlier exposed to 4 weeks of streptomycin had (near) normal audiograms, we did not attempt to enroll a matched control group that had not had streptomycin injections. Also, variability between measurements is likely to be higher than desirable, due to high levels of AN, although average levels of AN were only slightly higher compared to accepted bedside testing levels in the USA [34], [35]. This implies that the absolute numbers of hearing loss should be interpreted with caution, but as AN levels were similar for all measurements, it is not likely to have influenced the differences found between the two treatment arms.

The strength of our findings is that we could detect a difference between groups that appeared well matched. With known baseline characteristics, with a well-documented increased exposure to streptomycin, we conclude that prolonged streptomycin administration in the adult study subjects caused significant hearing loss, especially in the high frequency range. In addition, we obtained ototoxicity data from a patient sample that included both adults and children suffering from the same illness and receiving the same treatment, with differential effects in terms of toxicity.

Our study results support ongoing attempts to try replacing injected streptomycin by an alternative, e.g. clarithromycin. Currently a trial to compare standard care (streptomycin plus rifampicin) with oral treatment consisting of clarithromycin in extended release formulation combined with rifampicin is ongoing [36].

We conclude that streptomycin with cumulative dosages, especially in patients aged 16 or older should be given with caution, especially in individuals with concurrent risks for renal dysfunction or hearing loss.
